# Combined Locally Enhanced Electric Field Treatment and Copper for Effective Inactivation of Gram-Positive and Gram-Negative Bacteria in Water

**DOI:** 10.1021/acsestengg.5c00309

**Published:** 2025-07-25

**Authors:** Mourin Jarin, Jackie Ly, Alex Crowley, Shuyan Liu, Xing Xie

**Affiliations:** † School of Civil and Environmental Engineering, 1372 Georgia Institute of Technology, Atlanta, Georgia 30332, United States; ‡ School of Mechanical and Civil Engineering, 6469 California Institute of Technology, Pasadena, California 91125, United States

**Keywords:** LEEFT, Gram-negative, Gram-positive, copper, water disinfection

## Abstract

Locally enhanced electric field treatment (LEEFT) is an emerging technology that employs electric fields to inactivate bacteria in water. Compared to traditional chlorine-based solutions, LEEFT allows for efficient water disinfection while preventing the formation of harmful disinfection byproducts. When combined with copper (Cu), a material recognized for its antimicrobial properties, LEEFT-Cu has demonstrated increased bacteria inactivation efficiency. In this study, LEEFT-Cu is tested for its disinfection performance against 8 different bacteria (4 Gram-negative (G−) and 4 Gram-positive (G+)), each grown in both stable and exponential phases. The primary focus is on the effectiveness of LEEFT-Cu against both gram structures. It is concluded that LEEFT-Cu can achieve >3 log removal for most bacteria species (7/8) using <0.7 mg/L Cu. Additionally, the calculated degree of improvement using LEEFT-Cu in comparison to Cu ions alone indicates >20 times increase in disinfection performance. The degree of improvement also leads to the conclusion that G+ bacteria are up to 3 times more vulnerable to the impacts of EFT (i.e., increased membrane permeability) than G–. Future work should focus on testing the current bench-scale prototype with more complex water matrices to further advance LEEFT-Cu for practical applications in water disinfection.

## Graphical abstract

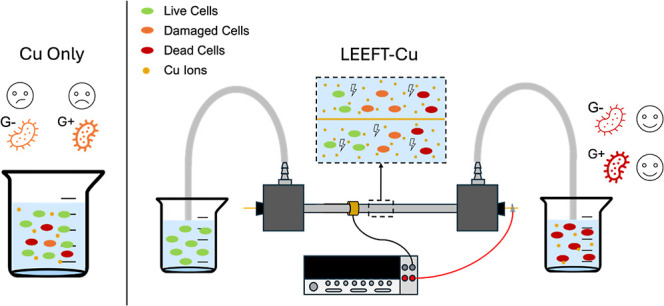

## 1 Introduction

The basic human right for access to clean and safe drinking water is currently still one of the most disregarded, as 25% of the world does not have consistent access to a safe drinking water supply.^1^ Water disinfection has been a necessary process to ensure safe drinking water for the population for more than 100 years.^2^ However, current chlorine-based water disinfection methods involve intense chemical usage and produce harmful disinfection byproducts (DBPs).^2,3^ These DBPs have been linked to increasing risks for the development of cancers and endocrine diseases.^4^ The consequence of using chemical disinfection has sparked interest in the usage of more physical disinfection techniques, including membrane filtration, UV, and electric field treatment (EFT) as they reduce/eliminate the formation of harmful DBPs.^5,6^ Recently, locally enhanced EFT (LEEFT) has emerged as a potential sustainable alternative to chlorine for primary/secondary disinfection. Previous work has shown LEEFT to achieve high inactivation with very low voltage, low energy consumption, and little or no side effects of treatment.^7,8^ LEEFT operates by enhancing an electric field either at the microscale or macro scale by configuration of the electrodes. LEEFT is distinct from electrochemical processes where inactivation is due to in situ-generated microbicidal chemicals (e.g., reactive oxygen and chlorine species) and/or direct electrochemical oxidation. While those electrochemical processes may still generate DBPs, LEEFT, in theory, will not change the physical and chemical properties of the treated water, i.e., generating DBPs.^9–13^ Its main method of inactivating pathogens has been reported previously as electroporation, althuogh some electrochemical oxidation may exist depending on the operating conditions.^14,15^

Previous works using LEEFT have tested different bacteria, both Gram-positive (G+) and Gram-negative (G−). In one study, LEEFT showed higher inactivation efficacy for *S. epiderimis* (G+) compared to *E. coli* (G−).^16^ While several other studies involving LEEFT have independently tested bacteria such as *E. coli* (G−), *S. enterica* (G−), *E. hormaechei* (G−), *E. faecalis* (G+), *B. subtilis* (G+), *E. durans* (G+), and *S. epidermidis* (G+), and showed G+ bacteria to be slightly more resistant, although the authors concluded the differences to be ultimately insignificant.^7,8,17,18^ Many authors have also studied the effects of EFT or pulsed electric fields on different gram-structure bacteria, and the findings vary on whether G+ or G– bacteria are more susceptible.^19–29^ Beyond this, LEEFT has also shown great results when combined with other disinfection methods or smaller dosages of chemical disinfectants like ozone or antimicrobial metals, specifically copper (Cu).^16,30,31^ Cu itself has been studied for its effects against G+ and G– bacteria previously, as the literature reports G+ bacteria to be more durable to Cu ions alone, attributed to the thicker peptidoglycan layer of the bacteria membrane.^32,33^

Additionally, LEEFT in combination with Cu (LEEFT-Cu) has previously reported significant results with >6 log inactivation when the EFT was combined with in situ Cu release.^30^ Our most recent work studying bacteria specifically introduced to both EFT and Cu elucidated that the relationship is significantly enhanced due to the synergistic effects of these two techniques.^31^ Our previous paper discussed the observation of the EFT weakening the cell membranes of *S. epidermis*, increasing the cell membrane permeability, and thus increasing the rate and ability for Cu ion uptake to the cells, ultimately leading to their death.^31^ This synergy was observed at the single cell level, showing high promise for the LEEFT-Cu reactor scale experiments as well. Despite this, these results are not directly translatable to the reactor scale, as they were accomplished on only one bacteria species, on an immobilized surface, and with no standard plate count methods used to confirm the ceiling of log inactivation efficiency. Therefore, no comprehensive study has been accomplished using the LEEFT-Cu system, specifically focused on gram differences.

The previous LEEFT-Cu work, although showing strong promise, was accomplished only using one species of bacteria and within only one growth condition.^30^ Previous works by many other authors in the field have also concluded that bacteria in different growth phases can widely vary in their susceptibility to Cu ions.^34–39^ To explore the practical applications of LEEFT-Cu, more complete information about its disinfection performance on a wide range of microorganisms must be gathered. Mainly, the goal was to explore the effectiveness of LEEFT-Cu across a wide range of bacteria to provide an in-depth understanding of inactivation performance, specifically regarding different membrane structures. Because of this, further investigation is needed for LEEFT-Cu for inactivation of different bacteria to understand whether the mechanism of inactivation is more synergistically enhanced with certain bacteria over others. In this study, the performance of LEEFT-Cu for 8 different G+ (4) and G– (4) bacteria was observed at various growth phases to better understand the LEEFT-Cu technology and physical mechanisms in which it is advantaged. Due to the literature understanding that Cu ions can have varied effects on different growth phases of bacteria and their resulting susceptibility to inactivation, cultures from exponential and stable growth phases were chosen for all bacteria tested.^34–39^ This was crucial to understand the effectiveness of LEEFT-Cu for applications in both early onset bacteria growth prevention (exponential/log phase) and disinfection capability for already matured (stable phase) bacteria. The results for the overall performance of LEEFT-Cu across the 8 different microorganisms, the log inactivation, the measured Cu concentrations, and the synergies analyzed are presented and discussed. Some insight into the impacts of these results and implementation of this technology is also highlighted, along with current limitations, challenges, and future directions for optimization.

## 2 Materials and Methods

### 2.1 Chemicals and Materials

Luria–Bertani broth (Miller, cat. no. 97064-114), Luria–Bertani agar (Miller, Culgene, cat. no. 89405-562), nutrient broth (Difco, cat. no. 234000), nutrient agar (Criterion, cat. no. C6461), tryptic soy broth (Millipore, cat. no. 22092), tryptic soy agar (Millipore, cat. no. 22091 and Wards’s Science cat. no. 470227-478), brain heart infusion broth (Bacto, cat. no. 237500), and brain heart infusion agar (Difco, cat. no. 241830) were purchased for bacteria growth and culture. Copper sulfate was purchased from Alfa Aesar for copper solution preparation. DI water (∼0.3 μS/cm) was collected from a Thermo Scientific Barnstead Nanopure system for all solution preparation and rinsing. Copper wire (∼255 μm diameter) was purchased from Arcor Electronics for the reactor electrode. Nitric acid (HNO_3_) (Sigma-Aldrich, cat. no. 225711) was used for sample preparation for the atomic absorption spectrometer. All bacteria were purchased from the American Type Culture Collection (ATCC), and their respective catalog numbers and species names, along with the growth conditions, are shown in Table 1. All 8 bacteria species were chosen for their accessibility in laboratory research and broad applicability to water and quality control testing. These 8 species include bacteria from different environments, contaminants, and soil. Using bacteria from various environments other than only traditional water pathogens was important to this study, as potable water contamination can result from multiple sources.

**Table 1:** Parameters for Each Bacteria Species and Its Growth Condition

bacteria species	ATCC no.	Broth and Agar	growth temperature (°C)	exponential growth phase selected (hours)	stable growth phase selected (hours)
*Escherichia coli* (G−)	10798	Luria–Bertani	35	4	15
*Citrobacter freundii* (G−)	8090	nutrient	35	4	15
*Alcaligenes faecalis* (G−)	8750	tryptic soy	35	16	20
*Enocbactor hormaechei* (G−)	700323	tryptic soy	30	5	15
*Staphylococcus epidermidis* (G+)	12228	nutrient	35	4	15
*Staphylococcus saprophyticus* (G+)	15305	nutrient	35	8	16
*Paenibacillus polymyxa* (G+)	842	nutrient	30	12	20
*Enterococcus durans* (G+)	6056	brain heart infusion	35	5	12

### 2.2 Fabrication of the LEEFT-Cu Device

Bench-scale tubular LEEFT-Cu devices were constructed as described in previous studies (shown in Figure S1).^40^ Briefly, reactors were fabricated by inserting a commercially available stainless-steel tube between two acrylic blocks with plugs on both ends. The stainless-steel tube itself measured 21.5 cm in length, and the inner diameter measured 4.88 mm, serving as the outer negative electrode, while the copper wire (diameter 255 μm) was firmly suspended within the hollow tube, serving as the center positive electrode. The total length of the reactor cylinder was 24.5 cm and resulted in a reactor volume of ∼5 mL. Both the outer and center electrodes were connected via conductive clips to a power source (Keithley 2400 SourceMeter). The breakdown of the inlet, outlet, and electrode placement is shown in Figure S1.

### 2.3 Bacteria Growth Curves and Influent Preparation

All bacteria cells were incubated at the temperature conditions recommended by the ATCC repository and with routine shaking of 200 rpm. Bacteria growth was monitored with consecutive measurements of each culture’s optical density (OD) at 600 nm wavelength with a UV–vis spectrophotometer (Hach, DR6000). This OD data was then plotted over time to produce the growth curves for each species tested (Figure S2). The specific times chosen for each species’ stable and exponential growth phases are listed in Table 1. Each species was cultured in their respective and recommended broth at the recommended temperature and conditions, resulting in bacterial concentrations of ∼10^7^–10^9^ CFU/mL after incubation. Important to note for the exponential growth phase bacteria concentration, they were often found to be about 1–2 orders of magnitude lower in cell count after incubation compared to the stable phase. This was expected, due to the shorter growth time in the exponential phase (when bacteria are multiplying the fastest). No significant impacts of this on the experiments or results were found, still allowing for the accurate calculations of the influent and effluent concentration through the standard plate count method for each condition. All bacteria were cultured aerobically, and the conditions for growth are also listed in Table 1 for each species. Once grown, all species were centrifuged three times with DI water 4000 rpm for 5 min using a VWR High Speed Microcentrifuge. The final washed solutions were then diluted 100-fold using prepared water or Cu solution samples to serve as the influent in all cases. The conductivity was measured using an Orion Versa Star Pro conductivity probe (Thermo Scientific). The conductivity of all influent solutions between 1.0 ± 0.4 μS/cm.

### 2.4 Bacteria Inactivation Experiments

An image of the experimental setup is represented, showing the influent, pump, reactor, effluent collection, and power source (Figure S1). For the Cu control experiments, CuSO_4_ solutions were made for 0.2, 0.4, 0.6, 0.8, 1.0, and 5.0 mg/L. Each concentration was verified using a CuVer 1 copper reagent test kit (Hach Method 8506, cat. no. 2105869) in the UV–vis spectrophotometer (Hach, DR6000). An aliquot of 100 μL of bacteria solution (∼10^7^–10^9^ CFU/mL) was suspended in 10 mL of Cu solution to achieve a bacteria concentration of ∼10^5^–10^7^ CFU/mL. Each Cu-bacteria solution was mixed thoroughly and left to sit for ∼2 h. After 2 h, each sample was diluted for plating. The 2 h period was chosen based on our previous work studying both EFT and/or Cu under the microscope, finding 2 h was an optimal period of time to enable Cu to perform successful inactivation while having no deviation from the results of EFT applied either independently or in combination.^31^

For each reactor experiment, 5 mL of washed ∼10^7^–10^8^ CFU/mL bacteria solution was diluted with 500 mL of DI water to create 500 mL of ∼10^5^–10^6^ CFU/mL bacteria solution influent. The operating voltage was chosen from preliminary experiments based off of previous LEEFT-Cu publications.^30^ The voltage was kept constant, and current was measured during experimentation. For an operating voltage of 1.5 V and flow rates of 1–5 mL/min, the current generally ranged from ∼50–140 μA. The constant voltage ensures a constant Cu release during operation of the LEEFT-Cu device, while the flow rate was used to adjust the hydraulic retention time (HRT), or time the solution is exposed to the center Cu electrode. The faster water flow reduces the residence time of water near the center Cu electrode. This limits the extent to which Cu ions accumulate in the water, leading to a lower overall Cu concentration in the effluent. HRT is equal to *V*/*Q*, where *V* is equal to the volume held by the reactor and *Q* is equal to the flow rate. Four HRTs were passed at each flow rate before sample collection in order to ensure consistency within the reactor experiments. The flow rate was set using a peristaltic pump (MasterFlex L/S). To ensure maximum uptake of Cu by the bacteria and consistency of control and reactor experiments, the effluent samples rested for 2 h before plating.

The initial concentrations of each bacteria and their different growth phases varied slightly (0.5–1.5 log difference on average), but based on the results, it does not affect the trends or conclusions of the data. This difference in the starting influent concentration is depicted clearly in the different detection limits for each species. Additionally, it is important to note that adsorption within the reactor setup typically accounted for up to ∼0.5 log removal in most experiments but did not take away from the results as it never accounted as the main mechanism of inactivation.

### 2.5 Analysis

Standard plate count methods were used for each respective agar to measure the bacterial concentration of the influent and effluent, and then log inactivation. All samples rested for 2 h before plating to ensure Cu uptake into the cells. The agar plates were incubated for 12–48 h (depending on bacteria species) at the respective growth temperatures listed in Table 1 to allow visible colony formation and ease of counting. Triplicates were used for all plate counts, and the standard deviation was calculated for all results. An Atomic Absorption Spectrometer (PerkinElmer PinAAcle 900F, with PerkinElmer S10 Autosampler) was used to measure the Cu release for all reactor experiments according to the USEPA method 7000B.^41^ Nitric acid was added to samples to create 2% nitric acid solutions for analysis by the AA Spectrometer. For all other data analysis, Microsoft Excel and standard two tailed *t*-tests were used to calculate p-values to assess significance. For *t*-test conditions comparing the two growth phases of bacteria, the values were paired, and for conditions where the gram structures were compared, the values from individual bacteria species were unpaired. The *t*-test for statistical significance is only provided for data sets with 8+ values; for data sets of <8 values, the statistical significance is not provided as the sample size was too small.

## 3 Results

### 3.1 Susceptibility of Bacteria to Copper Ions in Solution

The 8 different bacteria were all tested for their survival capacity using only Cu ions in solution to serve as a control to the reactor, which advantages both EFT and Cu. Figure 1 shows the log removal for varying Cu concentrations for all bacteria and their two tested growth phases. As expected, all the bacteria are somewhat susceptible to Cu in solution but to varying degrees as concentration increases. Generally, as the Cu concentration is increased, the log removal trends upward as well. When observing the difference between the stable and exponential growth conditions, it is clear for some bacteria like *E. coli* (G−) and *E. hormaechei* (G−), the exponential growth phase is more susceptible to Cu as the log removal increases more rapidly. For example, when observing *E. coli* (G−), Cu concentrations of 0.2 and 1.0 mg/L achieve 1.3 and 2.7 log removal in the stable growth phase but increase to 1.6 and 5.5 log removal for exponential growth phase. Similarly for *E. hormaechei* (G−), for Cu concentrations of 0.2 and 1.0 mg/L again, 0.3 and 1.1 log removal in the stable growth phase are observed, but these increase to 1.0 and 3.1 log removal for the exponential growth phase. For the other bacteria, like *S. epidermidis* (G+), *A. faecalis* (G−), and *E. durans* (G+), it is more difficult to conclude a difference, as their results mostly overlap. Almost no difference in susceptibility to Cu between the stable and exponential growth phases is observed. For *S. epidermidis* (G+), Cu concentrations of 0.2 and 1.0 mg/L observe only 0.1 and 0.6 log removal in the stable growth phase and remain fairly unchanged for exponential growth at 0.2 and 0.5 log removal. Similarly, *A. faecalis* (G−) is observed only 0.3 and 2.1 log removal for Cu concentrations of 0.2 and 1.0 mg/L in stable growth phase and remained fairly unchanged for exponential growth at 0.2 and 2.0 log removal. As well for *E. durans* (G+), the least susceptibility is observed out of all 8 bacteria, with only 0.01 and 0.10 log removal for Cu concentrations of 0.2 and 1.0 mg/L in the stable growth phase and remain extremely low for exponential growth as well at 0.02 and 0.11 log removal. It is clear that bacteria can vary on whether their growth phase creates more or less vulnerability to being inactivated by Cu.

**Figure 1:**
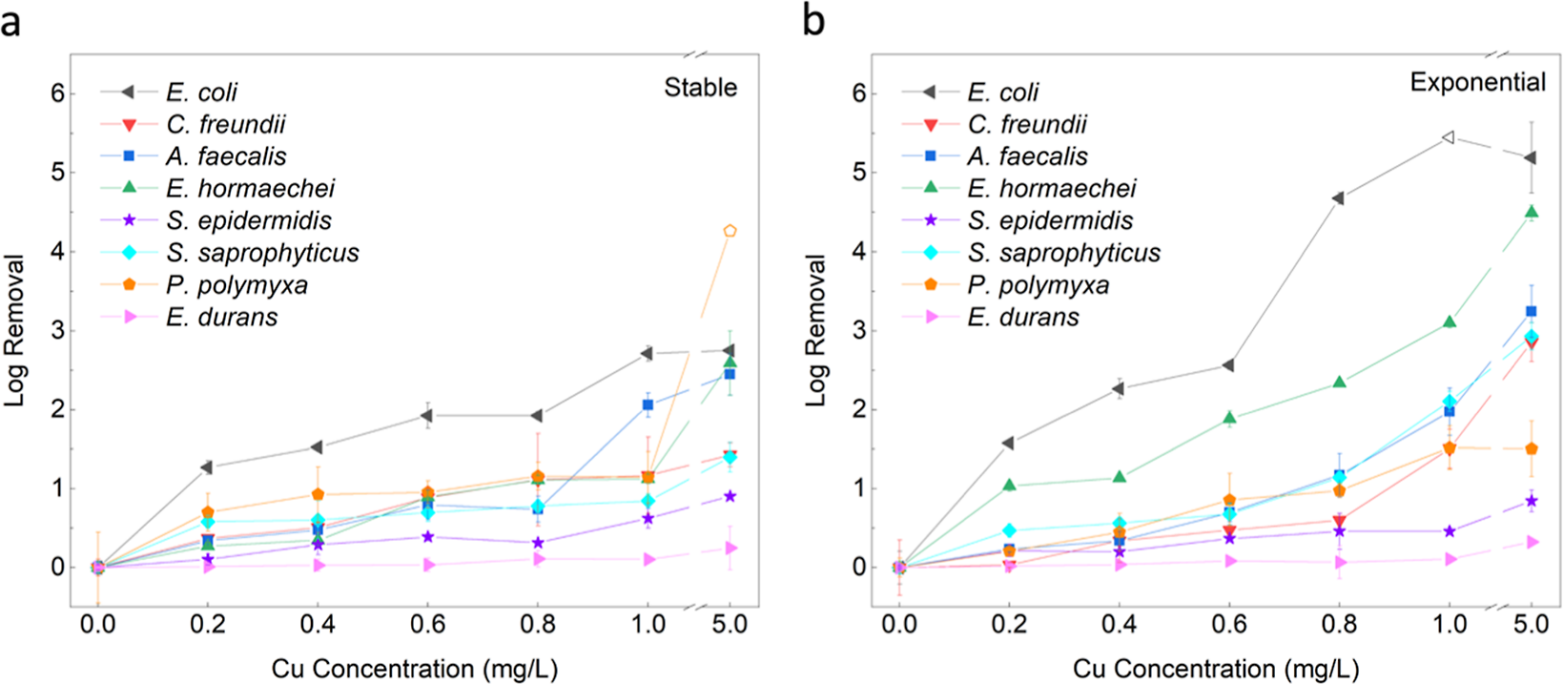
Susceptibility of bacteria to Cu ions in solution. The figures show the log removal per Cu concentration each bacteria in stable growth phase (a) or in exponential growth phase (b) for Cu concentrations 0–5.0 mg/L. Note the *x*-axis break from 1.0–5.0 mg/L. The error bars indicate the standard deviation from triplicate results. The hollow points depict values in which the detection limit was reached.

Interestingly, when testing with a concentration of 5.0 mg/L, most bacteria do not increase proportionally in cell death from 1.0 mg/L (Figure 1, *x*-axis break) as they did for the lower concentration range. The trend in almost all of the Cu control experiments is a very slow and gradual increase in inactivation at higher concentrations, and the amount of Cu ions is likely no longer of high importance. Instead, the Cu ion exposure time could be more important, as the longer the exposure bacteria have to Cu ions, the more likely they will die off. In our previous work studying EFT with Cu under the microscope, *S. epidermidis* (G+) was tested for its susceptibility to Cu ions over time from 0 to 180 min and found to increase linearly with time.^31^ Since each condition was tested for only 2 h exposure in this study, potentially longer exposure to Cu could aid in increasing the log removal as well. But for this case, it seems with only 2 h of exposure time, higher concentrations of Cu (1.0–5.0 mg/L) alone result in only a little additional log removal as concentrations increase.

Looking at Figure 1, there is no definitive observable difference between the growth phases or the gram structure of bacteria and their exposure to Cu ions. Because of this, the linear fit slope values of each bacteria were plotted to analyze and compare the log removal capacity per mg/L of Cu (Figure 2). Here, the susceptibility differences between stable and exponential growth phases are a bit clearer. For both stable and exponential growth phases, there is always a positive slope value, indicating the susceptibility to inactivation (log removal/Cu concentration) increases as Cu concentration increases. For *E. coli* (G−) specifically, the exponential phase susceptibility more than doubles from a value of 2.3 at stable to 5.3 for exponential. Additionally, the average susceptibility (log removal/Cu concentration) for all 8 stable growth phase bacteria was 1.1 ± 0.7, while for all 8 exponential growth phase bacteria, it was 1.9 ± 1.6 (almost double). Despite this, these two growth phases did not exhibit strong statistically significant differences in results when compared. Although, when considering the comparison between all the G– and G+ data, the average susceptibility (log removal/Cu concentration) for all 4 G– bacteria in both growth phases (4 stable and 4 exponential values) was 2.2 ± 1.4, while for all 4 G+ bacteria in both growth phases (4 stable and 4 exponential values), it was 0.8 ± 0.6 (almost 3 times less). These two groups also exhibited statistically significant differences in results when compared (*p* < 0.05).

**Figure 2:**
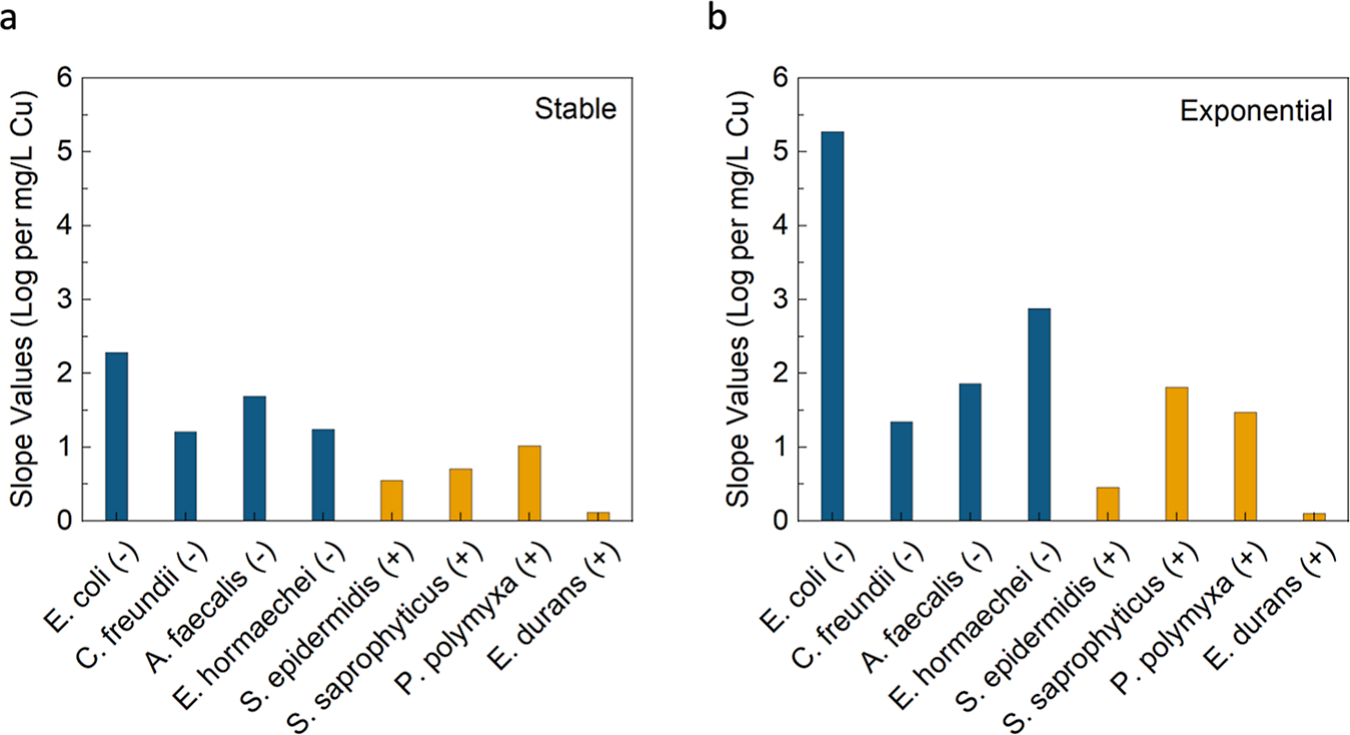
Slope values for linear fit lines from the trends shown in Figure 1 plotted for each bacteria species tested. (a) Stable growth phase. (b) Exponential growth phase. The linear fit lines are calculated for the values within 0–1.0 mg/L of Cu tested for each bacteria. The 5.0 mg/L values are not used for this calculation as the relationships within MCL guidelines were prioritized. There is also no statistical analysis provided as the data are analyzed and obtained only from the data points, trends, and relationships shown in Figure 1.

### 3.2 LEEFT-Cu Operation Using Varying Cu Concentration

The LEEFT-Cu reactor was tested for its ability to successfully disinfect and inactivate all 8 different bacteria using five different flow rates (1–5 mL/min), each resulting in a different HRT for the water passing through (further detailed in methods). The lower the flow rate, the longer the contact time of the water with the center Cu electrode, resulting in a higher effluent Cu concentration. Generally, the inactivation efficiency tends to decrease with increasing flow rate, as observed in previous LEEFT device studies.^7,8,42^ This is to be expected since the increased flow rate directly translates to less effective treatment time, lower Cu concentrations, and therefore lower inactivation results. Using the 5 different flow rates tested, the LEEFT-Cu reactor releases varying levels of Cu ions during operation. Figure 3 shows the log removal for all 8 bacteria for both growth phases relative to the Cu concentration measured. The log removal also typically increases with increasing Cu concentration using LEEFT-Cu, which is to be expected. All results have various ranges of Cu released from the reactor depending on the starting conductivity of the influent but generally range between 0.1 and 1.6 mg/L throughout the different operating parameters.

**Figure 3:**
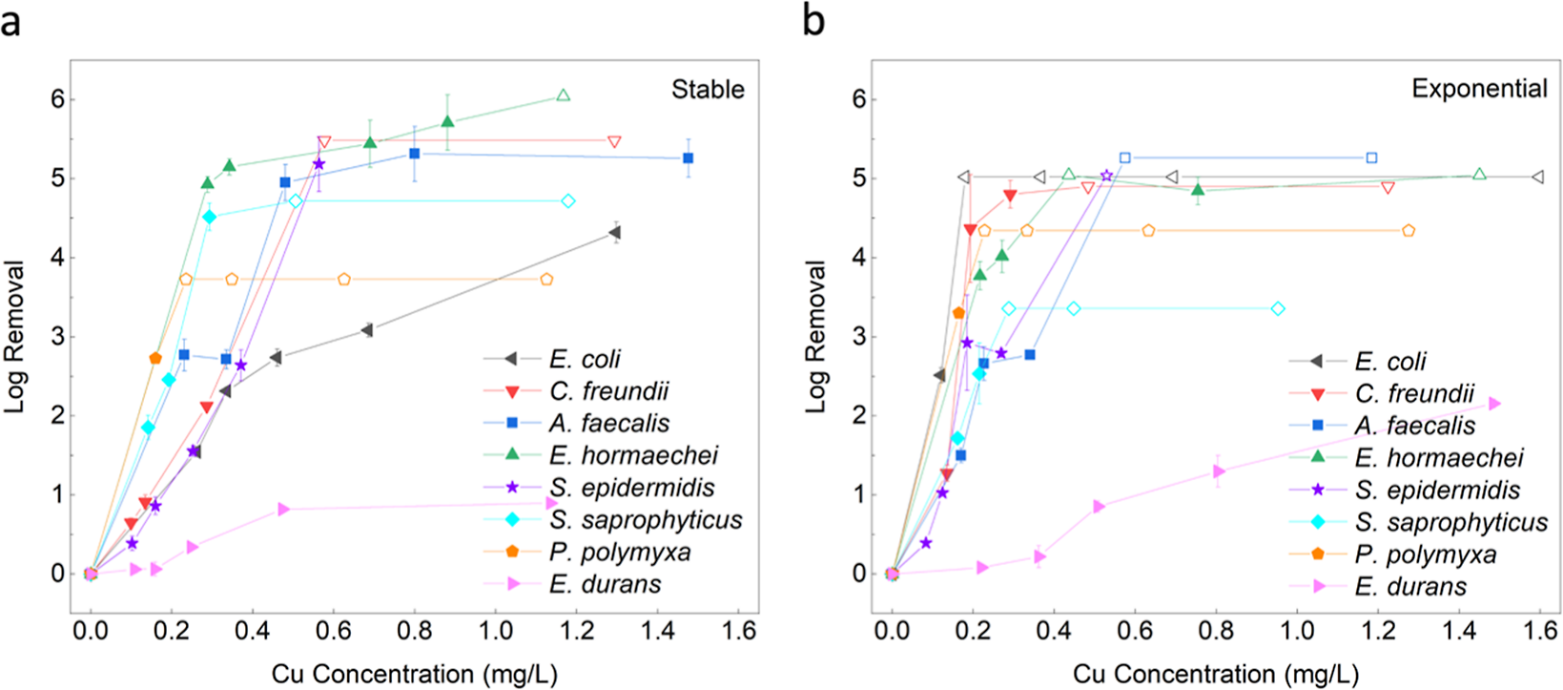
LEEFT-Cu results for log removal of 8 bacteria per Cu concentration released. (a) Stable growth phase. (b) Exponential growth phase. The error bars indicate the standard deviation from triplicate results. The hollow points depict values in which the detection limit was reached.

In most cases, the stable and exponential curves are rather similar in trend and log removal capacity. For example, for *A. faecalis* (G−), >5 log inactivation is achieved using only ∼0.50 mg/L Cu for the stable phase and ∼0.55 mg/L Cu for the exponential, showing no drastic differences. As for *S. epidermis* (G+), the stable growth phase observes >5 log inactivation with ∼0.55 mg/L Cu, and the exponential achieves this similarly with ∼0.53 mg/L Cu. For other cases, the bacteria show a clear difference in log removal between the stable and exponential growth phases. *E. coli* (G−), specifically, shows more susceptibility to LEEFT-Cu at the exponential growth phase, as it is able to reach >5 log inactivation with Cu concentrations of only ∼0.18 mg/L, while for the stable growth phase, it can only reach up to 4.3 log removal using 1.3 mg/L Cu. With all 8 bacteria tested, very high log removal can be achieved using LEEFT-Cu and ranging concentrations of Cu release. Looking closer, most bacteria (7/8 species) observed greater than 3 log inactivation with <0.7 mg/L Cu (with the exception of *E. durans* (G+)).

Looking at Figure 3, there is no definitive observable difference between the growth phases or the Gram structure of bacteria and treatment to LEEFT-Cu. Because of this, the linear fit slope values of each bacteria were plotted to analyze and compare the log removal capacity per mg/L of Cu when using the LEEFT-Cu reactor (Figure 4). Here again, the susceptibility difference between the stable and exponential growth phases is a bit clearer. For both stable and exponential growth phases, there is always a positive slope value, indicating that the susceptibility to inactivation (log removal/Cu concentration) increases as Cu concentration increases in LEEFT-Cu. For *E. coli* (G−), the exponential phase susceptibility increases almost 10-fold from a value of 3.1 for the stable phase to 26.9 for exponential. Additionally, the average susceptibility (log removal/Cu concentration) for all 8 stable growth phase bacteria was 7.4 ± 4.8, while for all 8 exponential growth phase bacteria, it is 12.6 ± 7.5. Despite the fact that the average susceptibility for both growth phases varied quite a bit, there were no statistically significant differences in results between them when compared. Furthermore, when considering the comparison within all the G+ and G– data, the average susceptibility (log removal/Cu concentration) for all 4 G– bacteria in both growth phases (4 stable and 4 exponential values) was 10.2 ± 7.4, while for all 4 G+ bacteria in both growth phases (4 stable and 4 exponential values), it was similarly 9.8 ± 6.3. These two groups also exhibited no statistically significant differences when compared.

**Figure 4:**
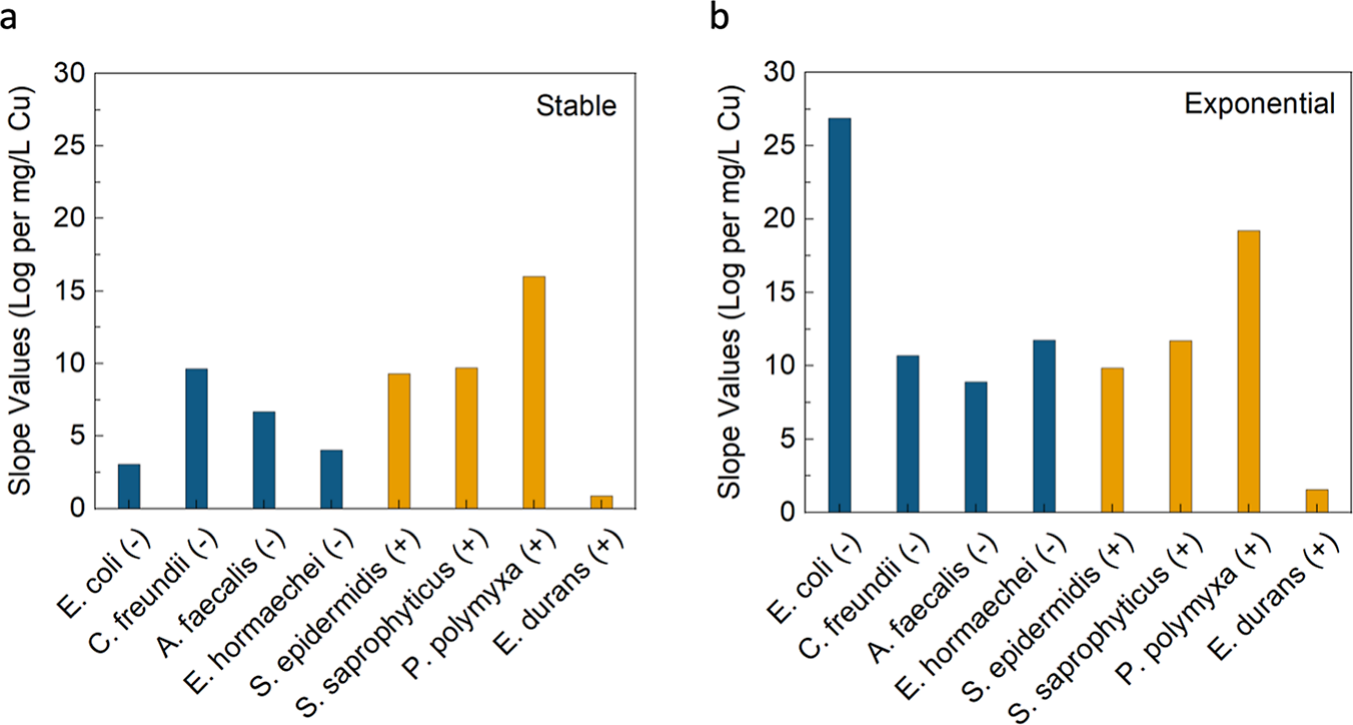
Slope values for linear fit lines from the trends shown in Figure 3 plotted for each bacteria species tested. (a) Stable growth phase. (b) Exponential growth phase. The linear fit lines for the LEEFT-Cu reactor results are calculated for values until the detection limit is initially reached. There is also no statistical analysis provided as the data are analyzed and obtained only from the data points, trends, and relationships shown in Figure 3.

### 3.3 Degree of Improvement Using LEEFT-Cu

The LEEFT-Cu and Cu only results for each individual bacteria tested are highlighted again more clearly in Figure S3. It is clear, looking at the wide gaps between the reactor data and the Cu controls, that for all 8 tested bacteria, the LEEFT-Cu system can achieve higher log removal and overall disinfection in comparison to Cu only solutions. When looking specifically at LEEFT-Cu’s ability to disinfect bacteria like *C. freundii* (G−), *A. faecalis* (G−), *E. hormachaechei* (G−), *S. epidermidis* (G+), *S. saprophyticus* (G+), and *P* *.* *polymyxa* (G+), it is clear there is up to a 3–5 log increase from when only Cu ions are used in the same concentration for both growth phases.

To further quantify the increase in disinfection performance for LEEFT-Cu in comparison to Cu ions alone, the degree of improvement was calculated and plotted for each bacterial species in Figure 5. Here, the degree of improvement is defined as the linear fit slope values from LEEFT-Cu (Figure 4) divided by the slope values for Cu only (Figure 2). For *E. coli* (G−) in the stable growth phase, the inactivation per Cu concentration improved by 1.3 times using LEEFT-Cu, and for the exponential growth phase, it increased its effectiveness by 5.1 times. For *S. epidermidis* (G+) in the stable phase, LEEFT-Cu improved the disinfection performance by 16.9 times using LEEFT-Cu and increased to 21.6 times more effective for the exponential phase. Additionally, the average degree of improvement for all 8 stable growth phase bacteria is 8.8 ± 6.0 times more effective using LEEFT-Cu, while for all 8 exponential growth phase bacteria, it is 9.8 ± 6.3 times more effective. These two growth phases exhibited no statistically significant differences in results when compared, indicating that despite the differences observed in previous sections between stable and exponential phase bacteria, LEEFT-Cu can improve the disinfection performance relatively equally among them. Lastly, when considering the comparison within all the G– and G+ data, the average degree of improvement for all 4 G– bacteria in both growth phases (4 stable and 4 exponential values) was 4.8 ± 2.3 times more effective, while for all 4 G+ bacteria in both growth phases (4 stable and 4 exponential values), it was 13.8 ± 5.0 times more effective (almost three times more). Notably, these two groups exhibited the most statistically significant differences when compared (*p* < 0.05).

**Figure 5:**
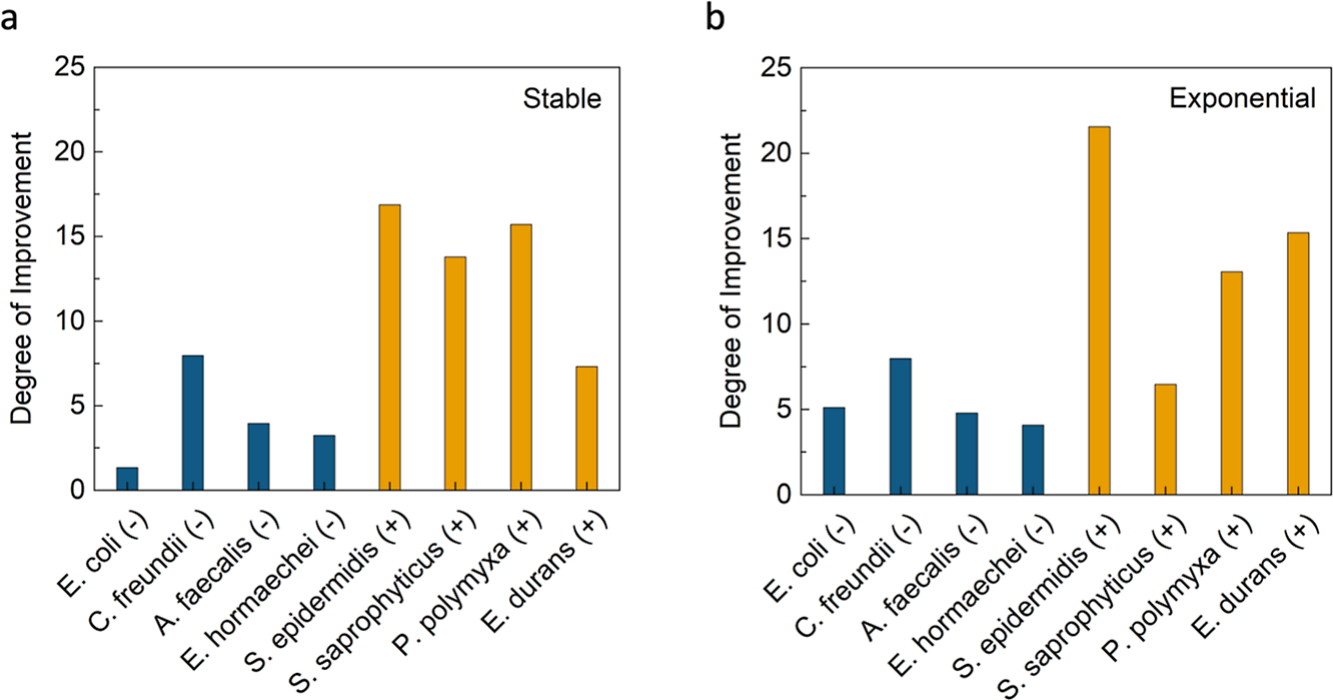
Plotted degree of improvement in log removal per Cu concentration of the LEEFT-Cu results in comparison to Cu only. (a) Stable growth phase. (b) Exponential growth phase. The degree of improvement is calculated by taking the ratio of the slopes obtained from the LEEFT-Cu results (Figure 4) divided by the slopes obtained from the Cu control results (Figure 2). The slope has no units here as it is a calculated ratio of two numbers with the same unit. There is also no statistical analysis provided as the data are analyzed and obtained only from the data points, trends, and relationships shown in Figures 2 and 4

## 4 Discussion

### 4.1 Resistance to Cu Inactivation

In this study, the LEEFT-Cu reactor was used to test its disinfection performance for 8 different bacteria species (4 G– and 4 G+) under both stable and exponential growth phases. These initial findings concluded some significant differences in log removal difference between stable and exponential bacteria (Figures 1 and 2) as stable cells were generally more resistant to inactivation by Cu ions alone. This difference in vulnerability is well established in the literature as, for example, Song et al. tested Cu ions for their ability to inactivate *L. pneumophila* (G−), finding exponential phase cultures to be 2.5 times more susceptible to Cu toxicity compared to stationary phase.^34–39^ This, along with the results, reassures us that on average, exponential growth phase bacteria can be almost twice as susceptible to inactivation by Cu ions than stable phase.

Additionally, the results showed significant statistical (*p* < 0.05) and log removal differences (almost 3 times less for G+) between G– and G+ bacteria using Cu ions alone (Figures 1 and 2). This leads us to believe that G+ bacteria are generally less susceptible to the inactivation by Cu. This is also well supported by the literature, as Cu ions have many well-known attack pathways to inactivate bacteria, specifically its abilities to cause protein dysfunction, cause membrane damage, and produce intracellular reactive oxygen species (ROS).^31,43–48^ Because of this, it is likely the thicker peptidoglycan layer of the G+ bacteria is serving as a protective shield to many of Cu’s attack pathways, especially those associated with the membrane impairment, leading to lower overall inactivation and log removal. Several authors also report G+ bacteria having higher resistance to common disinfectants chlorine, hydrogen peroxide, hydroxylamine, ultraviolet radiation, ozonation, hydrodynamic forces, and antimicrobial metals including silver and Cu, and attribute this to the thicker peptidoglycan layer as well.^28,32,49–54^ Specifically, Kim et al. studied Cu ions in combination with hydrogen peroxide and hydroxylamine for their inactivation capabilities on 5 G– (*E. coli*, *V. harveyi*, *S. flexneri*, *S. typhimurium*, and *P. aeruginosa*) and 3 G+ (*S. Pneumoiae*, *B. Subtilis,* and *S. Aureus*) bacteria.^32^ The authors concluded that G+ bacteria displayed lower inactivation by Cu­(II)-based combined microbicides due to the thicker peptidoglycan layer in the cell membrane.^32^ They also stated the thin layer of peptidoglycan in the cell membrane of G– bacteria aided in the facilitation and penetration of microbicides into the cells, leading to more damage.^32,33^ This work directly supports the Cu control results, showing significantly higher resistance in G+ bacteria compared to that in G–.

### 4.2 Degree of Improvement by LEEFT

This work was focused on how well LEEFT can aid in the inactivation efficiency achieved for both varying growth phases and Gram structures of bacteria. The degree of improvement calculated in comparison to Cu ions alone indicated that the contribution of LEEFT was equivalent to the total enhancement of log removal. The initial findings concluded there were no significant differences in the degree of enhancement between stable and exponential phase bacteria (Figure 5). For one case of these results, the log removal increased to over 20 times compared to that of Cu ions alone. Although LEEFT alone may achieve very little inactivation efficiency under the operating parameters used in this study, the likely permeability increase it causes to the membranes of both G+ and G– bacteria clearly improves the disinfection performance significantly by allowing Cu ions to take further advantage of their biocidal properties.^30^ Characterizing the LEEFT degree of improvement by gram structure, the effectiveness was found to be significantly more prominent in G+ bacteria than in G– bacteria (Figure 5). This indicates that the single set of lipid bilayer membranes of G+ bacteria may potentially be more susceptible to increased membrane permeability impacts by EFT than the double sets present in G– cells.

Interestingly, when looking at recent studies focused on EFT, the perspectives on whether the gram structure influences EFT results are quite diverse. A study by Zhang et al. using both chemical and physical disinfection methods discussed the contributing differences that help both *E. coli* (G−) and *S. aureus* (G+) bacteria resist electroporation.^24^ For the G– case, strong resistance to electroporation was mentioned to be because of the outer membrane’s limited mobility of lipopolysaccharide (LPS) molecules.^24,29^ On the other hand, the authors also stated the added protection of the thick and firm peptidoglycan wall provides to the inner membrane against electroporation for G+ bacteria (specifically for its physical barrier, dielectric property, synthase, and autolysin).^24,25,27^ A study by da Silva et al., focused on low intensity electric field inactivation of both *S. aureus* (G+) and *E. coli* (G−) bacteria and reported a better inactivation performance against the G+ bacteria, which they alluded to be because of the absence of an outer membrane.^55^ Despite this, other studies reported the opposite, finding G+ bacteria to be more resistant and less susceptible to these treatments when compared to G–.^19–23,26,56^ Huo et al. studied low-voltage electroporation applied to *E. coli* (G−), *E. faecalis* (G+), and *B. subtillus* (G+), finding G+ bacteria to require slightly larger doses in order to be killed effectively.^19^ Shawki et al. investigated the effects of DC electrical energy on G+ and G– bacteria strains (*P. Aeruginosa* (G−), *E. coli* (G−), *S. aureus* (G+), and *E. faecalis* (G+)) and found that the G+ species generally displayed less susceptibility against their electric strategies.^20^ Martens et al. studied the inactivation disparities between *Lactobacillus acidophilus* (G+) and *E. coli* (G−) bacteria, finding G– bacteria to be more vulnerable to electroporation due to the thinner peptidoglycan layer within the cell wall.^23^

It is clear from these studies that the effects of EFT can vary on the result of different gram structured bacteria; however, based on the degree of improvement results, it is clear that G+ bacteria are up to 3 times more vulnerable to the impacts of EFT, i.e., increased membrane permeability. One possible reason other studies using EFT are reaching such wide-ranging conclusions could be due to the potential side reactions in different experiments. Since the focus is on the application of EFT, the operating parameters in this study and previous ones have been chosen carefully to minimize potential side reactions like ROS generation, bubble formation, and any potential heating effects during operation.^31^ Even so, it can be quite difficult to control. Because of this, it is possible that for other studies, specifically those finding G– bacteria to be more susceptible to EFT, they may have introduced ROS or other side reactions into their experiments, leading the results to represent multiple mechanisms of disinfection rather than electroporation alone.

### 4.3 Effectiveness of LEEFT-Cu

With the data collected in this study and our previous work, we are now confident in the two main mechanisms of LEEFT-Cu to inactivate both G– and G+ bacteria in water. The first is the electric field exposed to the cells within the reactor that is well established in our previous works.^7,30,40^ The second mechanism is, of course, the Cu ions released during operation. As LEEFT uses local enhancement of the electrode through the coaxial configuration, the electric field is strongest at the center of the pipe.^14,30^ This electric field has been confirmed in our previous work to cause increased membrane permeability in the cells, and when strong enough, cause electroporation and cell death.^31,57^ Interestingly, previous papers have observed that the electric field strength necessary for inactivation of the cells using only EFT is ∼5–20 kV/cm, depending on the cell size and pulse parameters.^58,59^ Additionally, the contact time required to trigger electroporation and/or increased cell permeability is quite rapid (as quick as 20 ns in some previous cases).^57^ As all experiments in this study have a contact time of 1–5 min with the electric field, we are confident all the bacteria have had similar impacts from LEEFT alone. As the voltage application (and therefore produced electric field strength) is quite low in this study, it is highly likely the main inactivation mechanism for both the G– and G+ bacteria is a combination of increased cell membrane permeability, due to exposure to the electric field, and then ultimately, death by Cu ion uptake.^31^

Despite Cu ions alone showing a disparity in disinfection performance based on the gram structure (discussed previously, Figures 1 and 2), this ensures again that LEEFT-Cu is able to increase the membrane permeability and enhance the efficiency of Cu’s natural biocidal properties to close this gap and promote successful inactivation of both G+ and G–species. Ultimately, there is a strong level of inactivation improvement observed when using LEEFT-Cu at the bench scale compared to Cu ions alone, up to 20 times more effective depending on the bacteria species present (Figures 3 and 4), enabling the decrease of the Cu concentration necessary for inactivation. As this synergy is now closely observed at the LEEFT-Cu reactor scale, the combination of LEEFT with Cu ions are successfully able to disinfect both G+ and G– bacteria using low voltage and low Cu concentrations as predicted in our previous work.^31^ Lastly, it is very promising that LEEFT-Cu can not only significantly enhance the overall inactivation in comparison to Cu ions alone but also does so extremely effectively for both varied growth phases and gram structures.

### 4.4 Additional Advantages, Limitations, and Future Work

In comparison to LEEFT alone, the Cu ions in LEEFT-Cu provide a distinct advantage, as they provide a residual disinfectant for long-term use in water. Cu usage here also has several advantages to traditional disinfectants like chlorine. Cu in solid form is much more stable with a higher density, allowing the volume needed for transport to be reduced tremendously.^30^ Additionally, previous research on the LEEFT-Cu device estimated long-term disinfection of *E. coli* at 1.5 V and 1 min HRT, totaling a material cost of only $0.1/m^3^, which is similar to the cost of other municipal water treatment processes currently.^30^ Despite the advantages, the technology still does not come without a few limitations and challenges. First, the device is still mainly used as a lab scale prototype, with a holding volume of only 5 mL. The flow rates tested in this study range from 1 to 5 mL/min, which is rather low for some larger-scale applications. Because of this, further research should target larger scale device fabrication to enable higher flow rates for increased effluent volume. Future research should also involve methods that allow for further investigation and confirmation of the hypothesized mechanism involved in inactivation of the different pathogens that has not been previously observed at the reactor scale. These should include methods like membrane polarization tests to confirm how cell walls are impacted, microscopy imaging such as TEM to confirm similar mechanisms, or a linear scan voltammogram using a potentiostat to confirm no unwanted side reactions are present during voltage application. Lastly, the LEEFT-Cu device was tested in this study for 8 bacteria species with different gram structures but should also be tested in the future using higher and lower temperature waters, different water matrices, and over longer-term operation. Longer operation studies should also test solutions of mixed microbial contaminants in order to determine the performance and success of more realistic waters containing multiple species. Additional work should specifically target the efficacy of the device in more environmentally relevant samples like groundwater/surface water or simulated samples with similar conductivity. The device should also be tested for successful inactivation of viruses and algae as it is scaled up for more practical applications. Nevertheless, the results shown in this study for both G– and G+ bacteria as well as the success in inactivating both exponential and stable growth phases, show great promise for LEEFT-Cu to provide both a preventive measure and a solution for matured contamination water issues in the future.

## 5 Conclusion

In this study, the LEEFT-Cu reactor was used to test disinfection performance for 8 different bacteria species (4 G– and 4 G+), each grown in both stable and exponential phases. Emphasis was placed on achieving a wide range of data to conclude the effectiveness of LEEFT-Cu on different gram structured bacteria. After analyzing the LEEFT-Cu results in comparison to the Cu controls, we conclude LEEFT-Cu can achieve >3 log removal of most bacteria (7/8) species using <0.7 mg/L Cu. Additionally, statistically significant differences were observed between gram structures using only Cu ions, and no significance was observed between both growth phases and gram structures using LEEFT-Cu, as it was able to inactivate all bacteria almost equally. The calculated degree of improvement using LEEFT-Cu in comparison to Cu alone indicated that up to 20 times increase was possible in disinfection performance. A significant difference in degree of improvement was observed between G+ and G– bacteria, leading to the conclusion that G+ bacteria are more vulnerable to the impacts of EFT, i.e., increased membrane permeability. With the success of this work, it enables LEEFT-Cu to decrease the Cu concentration necessary for inactivating bacteria and provide a better solution to disinfecting drinking water. Future research should focus on maturing the current bench-scale reactor and testing more complex water matrices to further advance the LEEFT-Cu technology for practical applications in water disinfection.

## Supplementary Material



## Notes

Photo of experimental setup, including pump, LEEFT-Cu device, power source, and influent and effluent collection; growth curves of all 8 bacteria through measured absorbance over time; LEEFT-Cu experiments for all 8 individual bacteria for both growth phases relative to the Cu concentration measured (PDF)
