# Supplementary material for: Combined Locally Enhanced Electric Field Treatment and Copper for Effective Inactivation of Gram-Positive and Gram-Negative Bacteria in Water
Source: ACS ES T Eng. 2025 Jul 25;5(11):2833–43. doi: 10.1021/acsestengg.5c00309 (PMC12624728; doi:10.1021/acsestengg.5c00309)
Supplement: Supplementary file 1 [file ee5c00309_si_001.pdf]

Supplemental Information for

Combined Locally Enhanced Electric Field Treatment and Copper (LEEFT-Cu) for Effective Inactivation of Gram-Positive and Gram-Negative Bacteria in Water

Mourin Jarin[1], Jackie Ly[1], Alex Crowley[1,2], Shuyan Liu[1], Xing Xie[1*]

[1]School of Civil and Environmental Engineering, Georgia Institute of Technology, Atlanta, Georgia 30332, United States

[2]School of Mechanical and Civil Engineering, California Institute of Technology, Pasadena, California 91125, United States

[*]Corresponding author: Xing Xie, Email: xing.xie@ce.gatech.edu, Phone: 4048949723

1. Supplementary Figures

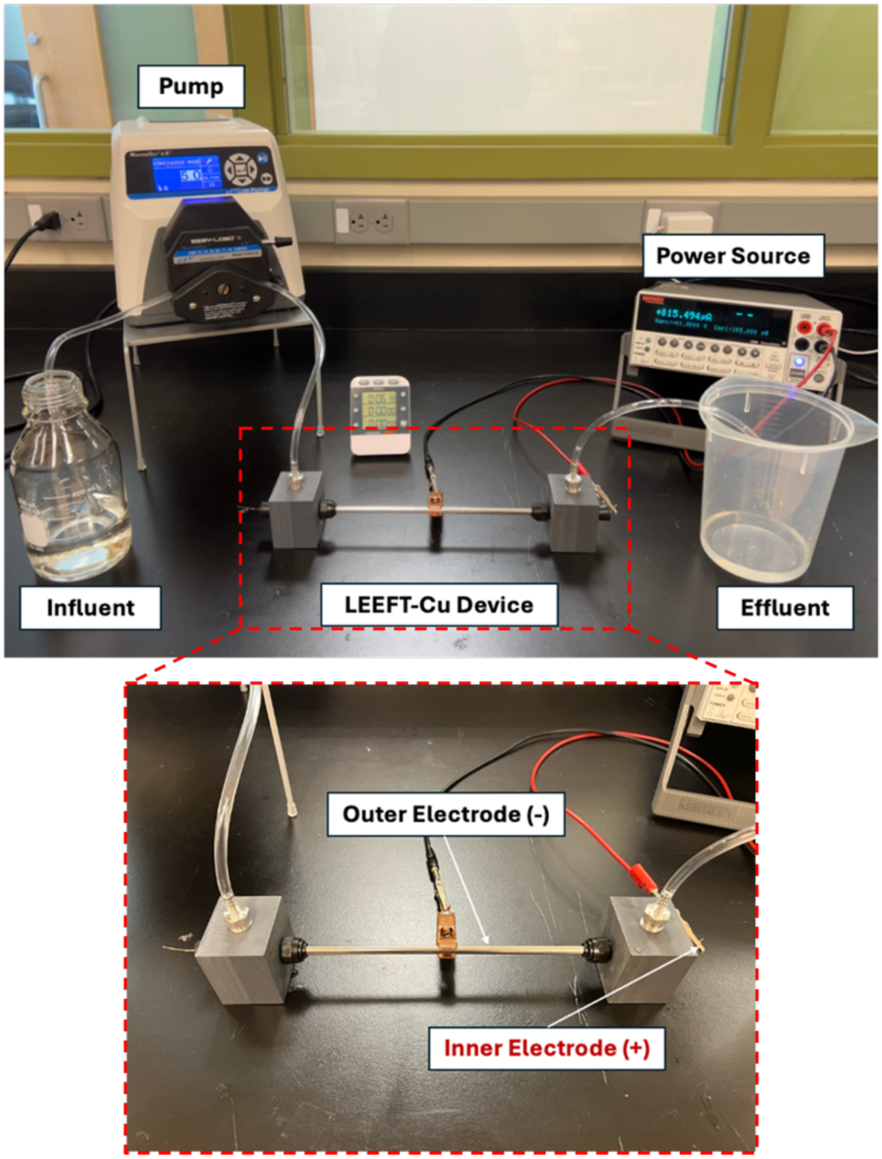

Figure S1. Photo of experimental setup including pump, LEEFT-Cu device, power source, and influent and effluent collection. The red dashed outline indicates the reactor and a zoomed in photo is provided depicting the inner electrode (copper wire) which serves as a cathode here, and outer electrode (stainless steel pipe) which serves as the anode. For any references to colors in the figure the reader is referred to the online/web version of this article.

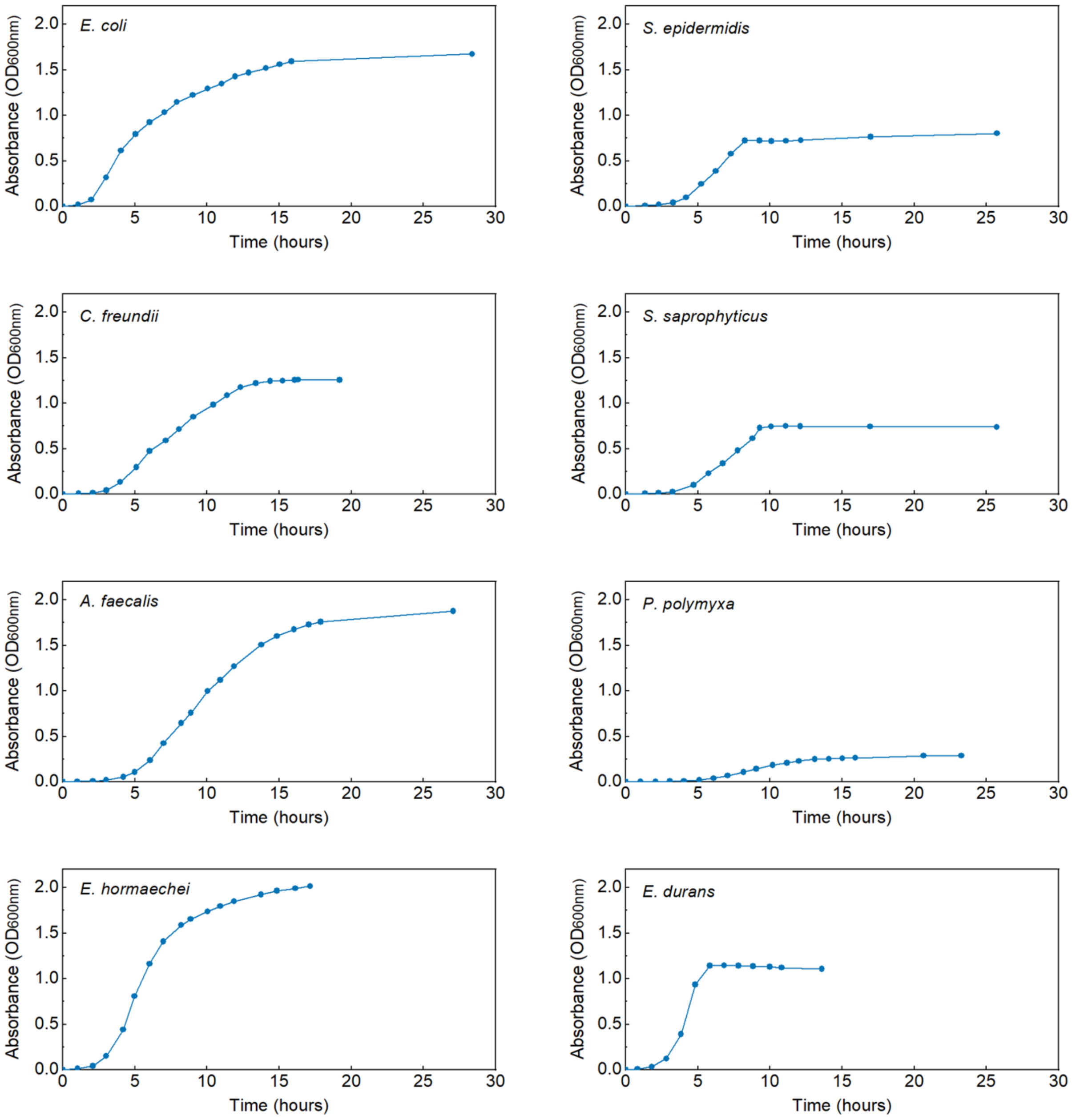

Figure S2. Growth curves of all 8 bacteria through measured absorbance (OD_{600nm}) over time. The left column depicts all the G- bacteria while the right column depicts the G+.

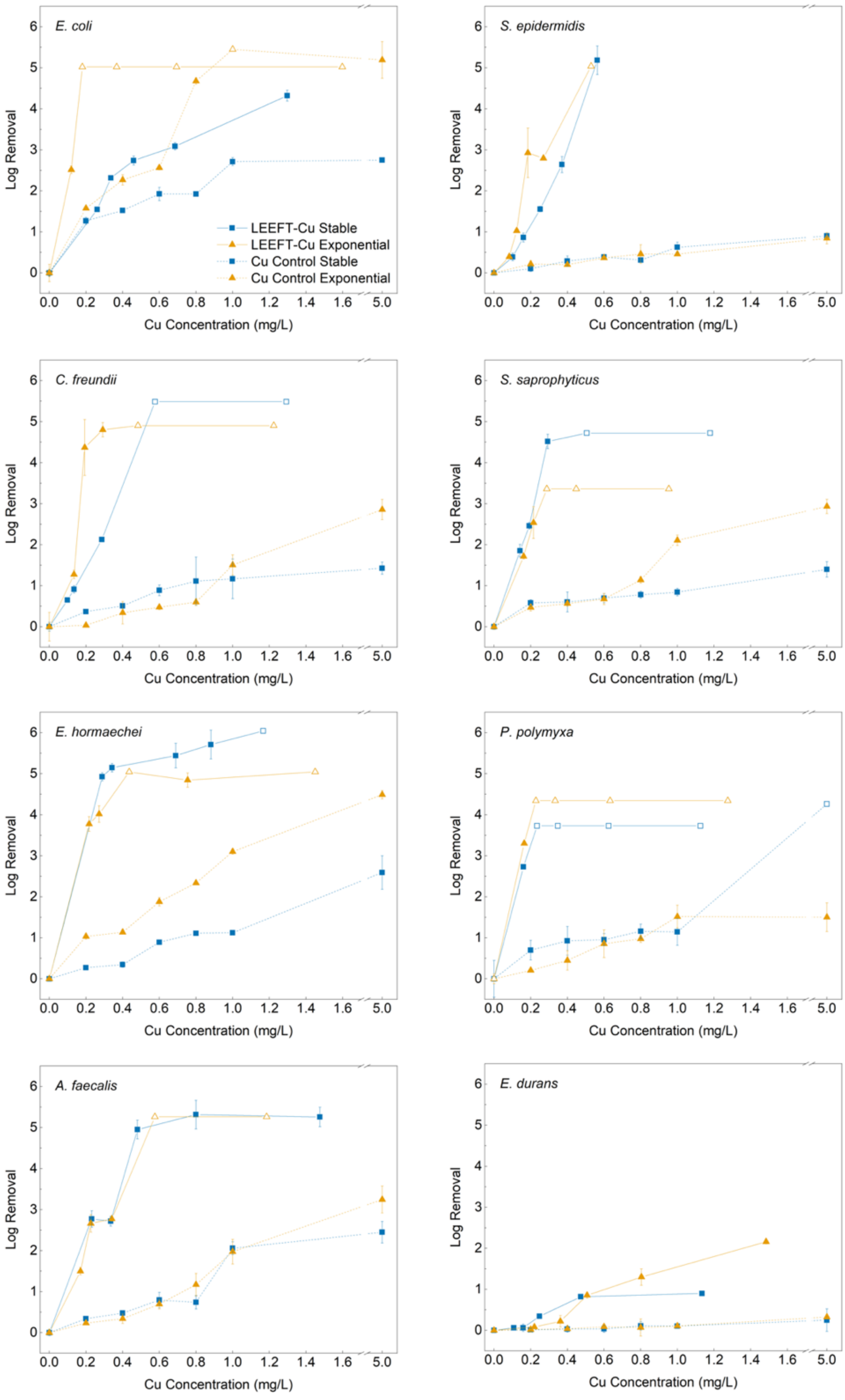
E. coli
S. epidermidis
C. freundii
S. saprophyticus
E. hormaechei
P. polymyxa
A. faecalis
E. durans
Log Removal
Cu Concentration (mg/L)
LEEFT-Cu Stable
LEEFT-Cu Exponential
Cu Control Stable
Cu Control Exponential

Figure S3. LEEFT-Cu experiments for all 8 individual bacteria for both growth phases relative to the Cu concentration measured. The Cu controls for each bacteria are also shown for reference. Note the x-axis break from 1.0 – 5.0 mg/L. The error bars indicate the standard deviation from triplicate results. The hollow points depict values in which the detection limit was reached.
